# Text Neck and Its Association With Cardiac Autonomic Function, Smartphone Addiction, and Psychophysiological Status in Young Adults

**DOI:** 10.7759/cureus.83025

**Published:** 2025-04-26

**Authors:** Divyasree Sureshbabu, Rajasegaran Rajalakshmi, Praveen Prakash, Sonit S Vasipalli

**Affiliations:** 1 Physiology, Jawaharlal Institute of Postgraduate Medical Education and Research, Puducherry, IND

**Keywords:** forward head posture, heart rate variability (hrv), neck disability index (ndi), nomophobia, text neck syndrome

## Abstract

Background

The increasing use of mobile devices has led to a rise in forward head posture (FHP), or "text neck," contributing to health issues. Additionally, nomophobia, or the fear of being without a mobile phone, has emerged as a growing behavioral concern, particularly among younger users.

Methods

This cross-sectional study aimed to assess FHP and its association with cardiac autonomic function, smartphone addiction, nomophobia, ergonomic risk, and neck disability in 84 participants aged 19-45 years. FHP was evaluated via craniovertebral (CV) angle measurement and cardiac autonomic status through heart rate variability, alongside smartphone addiction and psychological distress.

Results

Heart rate variability (HRV) analyses revealed a significant inverse relationship between the CV angle and the LF/HF ratio, indicating heightened sympathetic activity with reduced CV angle. Parasympathetic indicators, root mean square of successive differences between adjacent NN intervals (RMSSD) and pNN50, positively correlated with CV angle, suggesting a link between optimal posture and enhanced parasympathetic function. Psychophysiological assessments showed that smartphone addiction moderately correlated with nomophobia.

Conclusion

Text neck or forward head posture is associated with increased sympathetic and decreased parasympathetic modulation, as evidenced by HRV indices.

## Introduction

Neck pain, akin to low back pain, has escalated into a significant public health issue, largely driven by the increased usage of personal computers and mobile devices across all age groups [1]. This trend raises concerns as individuals frequently engage in prolonged texting and screen viewing, both of which contribute to the growing prevalence of neck pain [1].

Forward head posture (FHP), often referred to as “text neck,” is characterized by the forward protrusion of the head in relation to the trunk along the sagittal plane [2]. Studies have shown clear associations between FHP and the use of computing devices with screens, with the use of handheld devices in particular showing high degrees of FHP [2-4]. Forward head posture (FHP) has typically been observed in two broad forms. The first form involves cervical spine flexion, commonly seen when looking down at a smartphone. The second form is characterized by a combination of lower cervical flexion with upper cervical extension to maintain a horizontal gaze, which is often observed during computer use at a desk. The first form has been associated with a cluster of symptoms referred to as text neck syndrome (TNS), which includes headaches, neck discomfort, shoulder pain, arm pain, and back pain [2].

FHP can increase spinal disc pressure, raising the risk of disc herniation and degeneration. Due to the neuronal anatomy of the region, it may also affect the autonomic nervous system by disrupting sympathovagal balance, often leading to sympathetic dominance and heightened stress responses [5]. In FHP, hyperextension and forward translation of the cervical vertebrae can lead to C1-C2 misalignment [6]. This malalignment may compromise arterial circulation and restrict flow to cranial nerves that traverse this region, nerves that are responsible for regulating vital functions such as blood pressure and heart rate [7]. Neurons in the C1 region contribute to sympathetic excitation, and studies have shown that chemical stimulation of the rostral ventrolateral medulla activates sympathetic preganglionic neurons. This activation results in increased arterial pressure, elevated heart rate, and the release of adrenal catecholamines [6]. These findings provide a theoretical basis for how anatomical disruptions in FHP may influence cardiac autonomic function.

Text neck syndrome has been attributed to the repetitive strain resulting from FHP due to the overuse of handheld devices [8]. Beyond its commonly reported symptoms, text neck syndrome has also been associated with broader health concerns, including spinal misalignment, eye strain, dizziness, and frequent headaches. Additionally, the poor ergonomics linked to prolonged device use can lead to mental fatigue, negatively affecting mood and overall well-being. This research aimed to elucidate the prevalence of text neck among young adults and its relationship with cardiac autonomic and psychophysiological status, contributing to a more comprehensive understanding of the public health impact of text neck. Thus, the primary objective was to assess text neck (forward head posture) in young and middle-aged individuals (19-45 years) by measuring their craniovertebral (CV) angle using the photogrammetric measurement technique.

Besides FHP and TNS, another phenomenon related to smartphone use is nomophobia. Nomophobia (no-mobile-phone-phobia) refers to "the fear or anxiety individuals feel when they are without their mobile phone or unable to use it" [9]. This rising behavioral issue, driven by the widespread use of smartphones, is associated with symptoms like anxiety, emotional instability, irritability, and difficulty focusing. Research indicates that a substantial portion of smartphone users are affected by nomophobia, with younger individuals being more susceptible [9]. Thus, our secondary objectives included the assessment of the cardiac autonomic status (sympathovagal balance) of the study participants through the measurement of heart rate variability (HRV), the extent of smartphone addiction, and psychological status through the application of the self-reported Smartphone Addiction Scale-Short Version (SAS-SV) and Nomophobia Questionnaires, the level of ergonomic risk and disability using the Cornell Hand Discomfort (CHD) and Neck Disability Index (NDI) Questionnaires, and to determine the correlation between the above-mentioned parameters. To the best of our knowledge, the impact of text neck/forward head posture on cardiac autonomic and psychophysiological status in young and middle-aged individuals remains underexplored.

## Materials and methods

This cross-sectional study was undertaken in the Department of Physiology at the Jawaharlal Institute of Postgraduate Medical Education and Research (JIPMER), Puducherry, India, from April to July 2024. The study adhered to the ethical guidelines set by the Code of Ethics of the World Medical Association (Declaration of Helsinki 2013). All participants were informed about the study procedures, and written informed consent was obtained prior to their enrollment. The reporting of this study conforms to the Strengthening the Reporting of Observational Studies in Epidemiology (STROBE) guidelines.

Sample size estimation and sampling

The sample size was determined using the single proportion formula (OpenEpi, version 3; Atlanta, GA: Emory University). Based on a reported prevalence of 68% for text neck/forward head posture among college students, with a margin of error of 10% and a 5% significance level, a total of 84 participants were required [10]. Study participants who met the inclusion and exclusion criteria were recruited using a convenience sampling approach through flyers and campus-wide notices.

Inclusion and exclusion criteria

Institute employees, their relatives, and volunteers aged 19-45 years (both males and females) who self-reported using smartphones, laptops, computers, or tablets for at least 3 to 4 hours per day for more than one year, and who provided written informed consent, were included in the study. Individuals with a known history of (i) acute or chronic medical disorders, (ii) congenital postural deformities or pre-existing cervical pathologies, (iii) degenerative or inflammatory cervical spine disorders, (iv) cervical trauma, radiculopathy, or any previous surgeries involving the head, face, cervical spine, or limbs, (v) physiotherapy within the past three months, or recent use of analgesics, anti-inflammatory drugs, or muscle relaxants in the previous week, (vi) rheumatological or metabolic conditions, and (viii) pregnant or breastfeeding women were excluded from the study.

Procedure and measurement of text neck or forward head posture by the photogrammetric method

The sociodemographic information of the study participants was gathered and recorded in a data sheet. The Text Neck or Forward Head Posture (FHP) was identified by measuring the craniovertebral (CV) angle of the participants with digital imaging using the photogrammetric technique [11].

Photographs of each participant were captured in a well-lit room against a plain-colored wall using a mobile phone camera (64 megapixels) positioned on a tripod stand 150 cm away. The height of the phone was adjusted to align with the shoulder level of the subject. Adhesive markers were placed on the C7 spinous process and the tragus of the ear. Participants were instructed to stand sideways, with their left shoulder facing the camera and their gaze directed straight ahead. They were asked to flex and extend their head three times before resting it in a neutral, comfortable position. A lateral view photograph was then taken with the mobile camera. The images were transferred to a computer and analyzed using Kinovea software [12].

The craniovertebral (CV) angle was measured by calculating the angle formed at the intersection of a line connecting the midpoint of the ear's tragus to the skin over the C7 spinous process and a horizontal line passing through the C7 spinous process. In a systematic review by Mylonas et al., the photogrammetric method showed good to excellent intra-rater and inter-rater reliability and high criterion validity, provided the measurement process was standardized [13]. Participants with a CV angle of less than 50 degrees were classified as having a forward head posture [14].

Assessment of cardiac autonomic status (sympathovagal balance) by heart rate variability (s-HRV) technique

Cardiac autonomic status was evaluated using the short-term heart rate variability (HRV) analysis method. A lead II electrocardiogram (ECG) was recorded for 5 minutes after the subjects had rested in a supine position for 15 minutes, using the BIOPAC MP 150 system (Goleta, CA: BIOPAC Systems, Inc.). For the short-term HRV (s-HRV) analysis, R-R intervals were calculated from the 5-minute ECG recording after filtering out the ectopic beats and noise. These R-R intervals were then fed into Kubios HRV version 2.0 software (Kuopio, Finland: Kubios Oy), and the HRV analysis was performed according to the guidelines established by the Taskforce of the European Society of Cardiology and the North American Society of Pacing and Electrophysiology [15]. Both time domain and frequency domain HRV indices indicative of sympathetic and parasympathetic activity were analyzed.

Time Domain Indices of HRV

In the time domain, the standard deviation of all normal-to-normal (NN) R-R intervals (SDNN) was used as a measure of total HRV, reflecting the influence of both branches of the autonomic nervous system. Indices specifically indicative of parasympathetic activity included the root mean square of successive differences between adjacent NN intervals (RMSSD) and the pNN50, which is the number of pairs of adjacent NN intervals differing by more than 50 ms in the entire recording, divided by the total number of all NN intervals and serves as a measure of parasympathetic activity.

Frequency Domain Indices of HRV

For the frequency domain analysis, RR interval data segments of 256 seconds underwent processing involving cubic-spline interpolation, detrending, and Hanning window application, followed by fast Fourier transformation using Welch's periodogram averaging to compute the power spectral density. From this, low frequency (LF) power, calculated by integrating the power spectral density within the range of 0.04-0.15 Hz, was considered to represent both sympathetic and parasympathetic activity, while high frequency (HF) power, obtained by integrating the power spectral density between 0.15 and 0.4 Hz, primarily reflected parasympathetic activity. Further frequency domain measures included LF normalized units (LF nu), determined by dividing LF power by the total power and seen as an indicator of sympathetic activity, HF normalized units (HF nu), determined by dividing HF power by the total power and representing parasympathetic activity, and the LF/HF ratio, calculated by dividing LF power by HF power and serving as an index of sympathovagal balance.

Normalized units, which show the relative values of each power component in relation to the total power minus the VLF component, can be used to measure LF and HF. The portrayal of LF and HF in normalized units highlights the balance between the two branches. Furthermore, normalization tends to reduce the impact of changes in total power on the values of LF and HF components and makes it easier to compare across individuals because absolute levels of total power exhibit very significant intra- and inter-personal variance.

Poincare Plot Analysis

The Poincare plot was generated using the RR tachogram. This plot provides a visual method for analyzing time series data to detect underlying patterns. It is a two-dimensional graphical representation that shows the relationship between consecutive RR intervals, with each interval plotted against the next.

The following two primary descriptors are often utilized for quantitative analysis: (1) standard deviation 1 (SD1), which represents the short-term, beat-to-beat fluctuations in RR intervals, indicated by the spread along the ellipse's minor axis. (2) Standard deviation 2 (SD2) reflects the long-term variations in RR intervals, depicted by the spread along the ellipse's major axis.

Assessment of smartphone addiction and nomophobia

The level of smartphone addiction among study participants was evaluated using the Smartphone Addiction Scale-Short Version (SAS-SV). This is a 10-item self-reported tool that uses a six-point Likert scale, with total scores ranging from 10 to 60. Based on the original SAS-SV guidelines, the cut-off score for identifying problematic smartphone use is ≥31 for males and ≥33 for females [16]. The SAS-SV showed good reliability and validity for the assessment of smartphone addiction [17].

The individual's fear of being unable to use their smartphone was evaluated using the Nomophobia Questionnaire (NMP-Q) [18]. This questionnaire consists of 20 items rated on a seven-point Likert scale, where 1 represents "totally disagree," and 7 represents "totally agree." The questions are categorized into the following four key areas: inability to access information, loss of convenience, inability to communicate, and loss of connection. The total score is calculated by summing the responses for all items, resulting in a score range from 20 to 140, with higher scores indicating a greater level of nomophobia.

Assessment of ergonomics and neck disability

The ergonomic risk level of study participants was evaluated using the Cornell Hand Discomfort (CHD) Questionnaire, a validated and reliable tool for assessing hand pain [19]. The questionnaire covers three domains as follows: pain experience frequency, pain discomfort, and pain interference. The total discomfort score is calculated by multiplying the frequency, discomfort, and interference scores. The maximum score for a single shaded area of one hand is 45, and the combined total score for the six shaded areas is 270.

The assessment of neck disability was done using the Neck Disability Index (NDI) Questionnaire, which consists of 10 items addressing neck pain (intensity) and the individual's ability to perform daily activities, including "personal care, reading, lifting, headaches, work, concentration, driving, sleep, and recreation." There are six possible responses for each question, which equate to scores ranging from 0 to 5. As a result, the pain-associated disability classification score ranges from 0 to 50 points, where 0 to 4 represents no disability, 5 to 14 represents mild disability, 15 to 24 represents moderate disability, 25 to 34 represents severe disability, and 35 to 50 represents complete disability [20].

The individual's self-perceived severity of neck pain was assessed using the Numeric Rating Scale (NRS). A 10 cm straight line, marked with numbers from 0 to 10, was drawn on a sheet of paper. Participants were instructed to indicate the number that best represented the severity of their neck pain. Higher numbers on the scale corresponded to greater pain intensity. Pain intensity was assessed both while the participant was at rest and after performing active movements of the cervical spine, including flexion, extension, rotation, and lateral inclinations [21].

Statistical analysis

The data were entered into an Excel spreadsheet (Redmond, WA: Microsoft Corp.), and statistical analysis was performed using the Statistical Package for Social Sciences (SPSS) version 19.0 (Armonk, NY: IBM Corp.). The distribution of categorical variables, such as gender, socio-demographic variables, etc., was expressed as frequency and percentages. The continuous variables, such as craniovertebral (CV) angle, HRV indices, Smart Phone Addiction Scale (SAS-SV) score, Nomophobia score, Cornell Hand Discomfort (CHD) score, Neck Disability Index (NDI) score, and Numeric Rating Scale (NRS) score, were expressed as mean with standard deviation or median with interquartile range (IQR) according to the normality assumptions. The normality of data was assessed using the Shapiro-Wilk test.

The linear relationship between CV angle and HRV parameters, SAS-SV, nomophobia, CHD, NDI, and NRS scores was assessed using Spearman's rank correlation coefficient test. All statistical analyses were carried out at a 5% level of significance, and p<0.05 was considered significant.

## Results

Eighty-four participants (47 males and 37 females), with a mean age of 20.77±3.11 years, were recruited for the study. The median (IQR) values of CV angle and NDI were 54.79 (52.78-57.88) and 0.1 (0.04-0.16), respectively, and forward head posture was observed in 10 participants (11.90%). Participants had a mean SAS-SV score of 32.20±8.94 and a Nomophobia score of 68.07±20.52. Median pNN50 was 30.4 (17.22-46.22), while the median LF/HF ratio was 0.748 (0.37-1.19), with total power measured at 2628.5 (1306.25-3932.25) (Table 1).

**Table 1:** Characteristics of study participants. SD: standard deviation; IQR: interquartile range; CV angle: craniovertebral angle; SAS-SV: Smartphone Addiction Scale-Short Version; NDI score: Neck Disability Index score; HRV: Heart rate variability; RMSSD: root mean square of successive differences between adjacent NN intervals; pNN50: percentage of NN50 count divided by the total number of all NN intervals; SDNN: standard deviation of normal‑to‑normal intervals; VLF power: very low-frequency power; LF power: low-frequency power; LF nu: low-frequency power normalized units; HF power: high-frequency power; HF nu: high-frequency power normalized units; LF/HF: ratio of low‑to‑high‑frequency power

Parameters	Values (n=84)
Age (years) (mean±SD)	20.77±3.11
Gender: male (frequency {%})	47 (55.95%)
Gender: female (frequency {%})	37 (44.05%)
CV angle (median {IQR})	54.79 (52.78-57.88)
Forward head posture (frequency {%})	10 (11.90%)
SAS-SV score (mean±SD)	32.20±8.94
Nomophobia score (mean±SD)	68.07±20.52
NDI score (median {IQR})	0.1 (0.04-0.16)
Time-domain indices of HRV	
Mean RR (ms) (median {IQR})	852.55 (746.68-919.30)
RMSSD (median {IQR})	55.7 (41.05-77.48)
pNN50(median {IQR})	30.4 (17.22-46.22)
SDNN (median {IQR})	55.75 (44.08-68.60)
Frequency domain indices of HRV	
VLF power (ms^2^) (median {IQR})	662.5 (412-1013.25)
LF power (ms^2^) (median {IQR})	609 (315-1097)
LF n.u. (median {IQR})	42.74 (26.95-54.39)
HF power (ms^2^) (median {IQR})	1050 (417.25-1817.50)
HF n.u. (median {IQR})	57.24 (45.54-73.05)
LF/HF (median {IQR})	0.748 (0.37-1.19)
Total power (ms^2^) (median {IQR})	2628.5 (1306.25-3932.25)

While a significant negative correlation was observed between CV angle and LF/HF ratio (ρ = -0.799, p<0.001), RMSSD and pNN50 were positively correlated with CV angle (ρ=0.399, p<0.001 and ρ=0.377, p<0.001, respectively). SD1 showed a significant positive correlation with CV angle (ρ=0.421, p<0.001). SDNN also showed a weak positive correlation with CV angle (ρ=0.220, p=0.018) (Table 2).

**Table 2:** Correlation between CV angle and various study parameters. *P<0.05 is considered statistically significant. ρ-values are Spearman's rank correlation coefficient. CV angle: craniovertebral angle; SAS-SV: Smartphone Addiction Scale-Short Version; NDI score: Neck Disability Index score; NRS: Numeric Rating Scale; RMSSD: root mean square of successive differences between adjacent NN intervals; pNN50: percentage of NN50 count divided by the total number of all NN intervals; SD 1: standard deviation 1; SD 2: standard deviation 2; SDNN: standard deviation of normal‑to‑normal intervals

Parameters	ρ-Value	p-Value
SAS-SV score	0.107	0.412
Nomophobia score	0.099	0.285
NDI score	-0.083	0.992
NRS resting	-0.003	0.816
NRS flexion	0.018	0.911
NRS extension	-0.097	0.732
NRS rotation	0.053	0.675
NRS lateral inclination	-0.039	0.416
Total power	0.181	0.061
LF/HF ratio	-0.799	<0.001*
RMSSD	0.399	<0.001*
pNN50	0.377	<0.001*
SD1	0.421	<0.001*
SD2	0.093	0.214
SDNN	0.220	0.018*

There was no significant correlation between the SAS-SV score and CV angle. However, a significant moderate positive correlation was observed between the SAS-SV score and nomophobia (ρ=0.468, p<0.001) and the SAS-SV score and the NDI score (ρ=0.427, p<0.001). Significant but weak positive correlations were observed between the SAS-SV score, NRS flexion (ρ=0.250, p = 0.016), extension (ρ=0.179, p=0.041), and rotation (ρ=0.226, p=0.010) (Table 3).

**Table 3:** Correlation between the SAS-SV score and various study parameters. *P<0.05 is considered statistically significant. ρ-values are Spearman's rank correlation coefficient. CV angle: craniovertebral angle; SAS-SV: Smartphone Addiction Scale-Short Version; NDI score: Neck Disability Index score; NRS: Numeric Rating Scale; RMSSD: root mean square of successive differences between adjacent NN intervals; pNN50: percentage of NN50 count divided by the total number of all NN intervals; SD 1: standard deviation 1; SD 2: standard deviation 2; SDNN: standard deviation of normal‑to‑normal intervals

Parameters	ρ-Value	p-Value
CV angle	0.107	0.412
Nomophobia score	0.468	<0.001*
NDI score	0.427	<0.001*
NRS resting	0.037	0.534
NRS flexion	0.250	0.016*
NRS extension	0.179	0.041*
NRS rotation	0.226	0.010*
NRS lateral inclination	-0.074	0.229
Total power	-0.064	0.752
LF/HF ratio	0.025	0.786
RMSSD	0.050	0.593
pNN50	0.041	0.719
SD1	0.049	0.608
SD2	-0.035	0.997
SDNN	0.015	0.670

## Discussion

Eighty-four young adults (47 males and 37 females) aged 20.77±3.11 years were recruited for this study. The forward head posture of the participants was assessed by measuring their craniovertebral (CV) angle using the photogrammetric measurement technique, and the mean CV angle of the participants was 54.79°. The cardiac autonomic status, the extent of smartphone addiction, psychological status, level of ergonomic risk, and disability were assessed by heart rate variability (HRV) technique, Smartphone Addiction Scale, Nomophobia Questionnaire, Cornell Hand Discomfort, and Neck Disability Index (NDI) Questionnaires, respectively.

The results of this study align with the theoretical understanding that posture can influence autonomic function. Poor posture, such as FHP, may lead to mechanical strain and altered proprioceptive feedback from cervical structures, potentially activating stress responses mediated by the sympathetic nervous system [22]. Previous studies have demonstrated that certain postural positions can affect autonomic balance. The study by Shaffer and Ginsberg reported that slumped sitting posture decreased parasympathetic activity compared to upright sitting [23].

The positive correlations between CV angle and parasympathetic markers support the fact that maintaining optimal head and neck posture helps maintain cardiac parasympathetic activity. Increased parasympathetic modulation is beneficial for stress reduction and cardiovascular health, as it promotes relaxation and recovery processes [23]. This suggests that interventions aimed at correcting FHP could have favorable effects on autonomic function.

The weak or non-significant relationships between CV angle and psychophysiological parameters like smartphone addiction, nomophobia, and neck pain indices indicate a complex interplay between posture and psychological factors. While excessive smartphone use has been associated with FHP and musculoskeletal discomfort [24,25]. The findings of this study suggest that CV angle alone may not directly mediate these relationships. Psychological factors such as anxiety and addiction might independently influence both device use and autonomic function, necessitating further research to unravel these associations. A study by Acharya et al. highlighted that smartphone addiction could alter HRV, indicating increased sympathetic activity, but the role of posture was not directly examined [26].

HRV total power, RMSSD, pNN50, SDNN, SD1, and SD2 are known to show significant interdependency, highlighting the coherence between these HRV indices. This coherence underscores the integrative nature of HRV metrics in reflecting the dynamic balance of the autonomic nervous system [27]. The present study also showed simultaneous correlations between CV angle and RMSSD, pNN50, SDNN, and SD1. The consistency among these measures suggests that they can be reliably used to assess autonomic function in relation to postural changes.

Clinical and ergonomic implications

The association between FHP and increased sympathetic dominance (LF/HF ratio) underscores the importance of ergonomic interventions to correct posture, particularly in populations with high smartphone usage. Ergonomic education, promoting awareness of proper head and neck alignment, and encouraging regular breaks during device use, may mitigate the adverse autonomic effects associated with FHP. Healthcare professionals should consider assessing posture as part of routine evaluations, especially for individuals presenting with stress-related symptoms or cardiovascular risk factors. Interventions such as physiotherapy, chiropractic adjustments, and specific exercises have been shown to improve FHP and may enhance HRV parameters [28,29].

Interventions aimed at improving posture could enhance parasympathetic activity and decrease sympathetic activity, offering potential benefits for stress management and cardiovascular health. Incorporating posture correction exercises and mindfulness practices into wellness programs may provide holistic benefits for autonomic regulation and overall well-being. Moreover, the use of wearable technology and biofeedback devices can assist individuals in monitoring and correcting their posture in real time [30].

Limitations and future direction

A methodological limitation was the use of photogrammetry for the assessment of FHP rather than the gold standard of radiographic measurement using a lateral X-ray of the neck, primarily due to resource constraints. Another limitation is the cross-sectional design, which does not allow for causal inferences to be made. Longitudinal studies are needed to determine whether improving CV angle leads to sustained changes in HRV and autonomic balance. Additionally, while we assessed smartphone addiction and nomophobia, other psychological factors, such as general stress levels and mental health status, which may influence the observed relationships, were not evaluated. Future research should consider these variables and investigate the potential mediating effects of posture on the relationship between psychological factors and autonomic function. Furthermore, the generalizability of our findings may be limited due to the homogeneity of the sample. Future studies could explore a more diverse sample or alternative methodological approaches to enhance generalizability.

## Conclusions

This study provides compelling evidence that forward head posture (FHP), commonly referred to as "text neck," is associated with imbalances in cardiac autonomic regulation, specifically, an increase in sympathetic nervous system activity and a reduction in parasympathetic modulation, as measured by heart rate variability (HRV) indices. These findings support the growing body of literature suggesting that poor postural alignment, particularly of the head and neck, can contribute to autonomic imbalance. This relationship underscores the importance of maintaining ergonomic alignment in daily activities, especially during prolonged smartphone use or desk work. Incorporating ergonomic education, posture correction exercises, and wearable feedback tools may offer simple, effective strategies to enhance both physical and autonomic well-being.
